# Severe chronic UTI sustained by clinically undetected intracellular *Escherichia coli* in a pediatric patient

**DOI:** 10.1128/asmcr.00077-25

**Published:** 2025-10-21

**Authors:** Arthika Manoharan, Naveen Wijekoon, Greg Whiteley, Theerthankar Das Ashishkumar, Neftali Flores-Rodriguez, Slade O. Jensen, Bill Söderström, Kate H. Moore, Jim Manos, Ben J. Marais, Aniruddh Deshpande

**Affiliations:** 1 Infection, Immunity and Inflammation Theme, School of Medical Sciences, Charles Perkins Centre, The University of Sydney, Sydney, Australia; 2 Sydney Infectious Diseases Institute (Sydney ID), The University of Sydney, Sydney, Australia; 3 Children’s Hospital at Westmead, The University of Sydney, Sydney, Westmead, Australia; 4 Whiteley Corporation, Tomago, Australia; 5 School of Medicine, Ingham Institute for Applied Medical Research, Western Sydney University, Sydney, Australia; 6 Sydney Microscopy and Microanalysis, The University of Sydney, Sydney, Australia; 7 Australian Institute of Microbiology and Infection, University of Technology, Sydney, Australia; 8 Department of Urogynaecology, St. George’s Hospital Kogarah, University of New South Wales 7800, Sydney, Australia; 9 Sydney Medical School, The University of Sydney 4334, Sydney, Australia; 10 Faculty of Medicine and Health, University of Newcastle 5982, Newcastle, Australia; Pattern Bioscience, Austin, Texas, USA

**Keywords:** urinary tract infection, UTI, intracellular bacterial communities, IBCs, uropathogenic *E. coli*, UPEC, pediatric, adolescent, chronic

## Abstract

**Background:**

The presence of intracellular bacterial communities (IBCs) in the urothelium has been well documented in adults with chronic urinary tract infections (UTIs), but its long-term persistence going undetected in a severely symptomatic adolescent has not been reported.

**Case Summary:**

We present the case of a 14-year-old girl suffering from debilitating chronic UTI symptoms and associated urinary incontinence for many years. Multiple antibiotic courses provided only temporary relief, with positive *Escherichia coli* cultures recurring promptly after each treatment cycle. A recently conducted cystoscopy (after >6 years of persistent symptoms) revealed widespread squamous metaplasia of the bladder wall, and enhanced urinary analysis identified extensive intracellular bacterial (*E. coli*) communities in exfoliated urothelial cells. These intracellular bacterial communities persisted even when the urine became transiently culture negative on antibiotic treatment. Evidence from confocal microscopy demonstrated extensive intracellular *E. coli*, which may serve as a bacterial reservoir that seeds urinary reinfection when antibiotics are ceased. Persistent intracellular bacteria were not detected by routine urine microscopy and culture. Analysis of urinary cytokines suggested chronic inflammation of the bladder wall, driven by persistent bacterial infection, as the potential cause for the unrelenting symptoms.

**Conclusion:**

This is the first report demonstrating long-term undetected IBC in a severely symptomatic child with chronic UTI. It underscores the need to learn more about intracellular bacteria and urinary tract biofilms that are protected from antibiotics and host immunity. IBC reservoirs seem to drive bladder wall inflammation, exacerbating clinical symptoms and increasing the risk of long-term adverse sequelae.

## INTRODUCTION

Urinary tract infections (UTIs) are responsible for a large and rising global disease burden, with over 400 million infections reported annually (1). Chronic UTIs are often defined as two or more microbiologically confirmed UTIs within a 6-month period (2, 3), but this fails to capture the full spectrum or corresponding pathogenesis of disease. Forty percent of children with UTIs will suffer recurrent episodes despite successful antibiotic treatment (4, 5), and many may meet the 6-month threshold to be classified as “chronic UTI,” with this subgroup causing major clinical concern. These are the children who develop persistent debilitating symptoms over an extended period that are not responsive to antibiotic therapy. The disease spectrum and underlying pathophysiology in this severely affected subgroup are poorly described.

In adult female patients, chronic UTI symptoms have been ascribed to the presence of intracellular bacterial communities (IBCs) that are biofilm-like and facilitate bacterial persistence despite antibiotic treatment (6, 7). IBCs exhibit classic biofilm properties, such as encasement in self-produced polymeric substances with enhanced resistance to phagocyte and antibiotic clearance (8, 9). IBC formation is primarily associated with uropathogenic *Escherichia coli* (UPEC) (7, 10). Although the occurrence of intracellular bacteria in pediatric patients has been recorded (11), its persistence and lack of detection by routine cultures in a single patient has not been documented before. We present the case of a young female suffering from UTIs for over 6 years with no resolution, in accordance with the CARE guidelines (12).

## CASE PRESENTATION

A 14-year-old girl presented with long-standing recurrent UTI episodes that coalesced into a continuous symptomatic state in the last 3 years. The patient experienced dysuria, urgency, frequency of urination, and severe daytime urinary incontinence in the context of struggling with recurrent UTIs since the age of 8.

Given ongoing symptoms over the past 6 years, mid-stream urine cultures sampled regularly (every 1–2 months) yielded both pan-susceptible and cephalexin-resistant *Escherichia coli* (≥10^8^ CFU/mL), without any prolonged period (≥6 months) of clearance. She received repeated courses of oral cephalexin (15 mg/kg), usually for 5–7 days or until urine cultures cleared. This was changed to either nitrofurantoin (100 mg/day) or co-trimoxazole (24 mg/kg) for 5 days if cephalexin-resistant strains were identified. She was gluten intolerant but otherwise healthy. No renal tract abnormality was detected on multiple ultrasound investigations. Given the frequency of UTI recurrence and their psychosocial impact on her day-to-day life, she was referred to the specialist pediatric urology unit at the Children’s Hospital, Westmead, Sydney, Australia.

**Fig 1:**
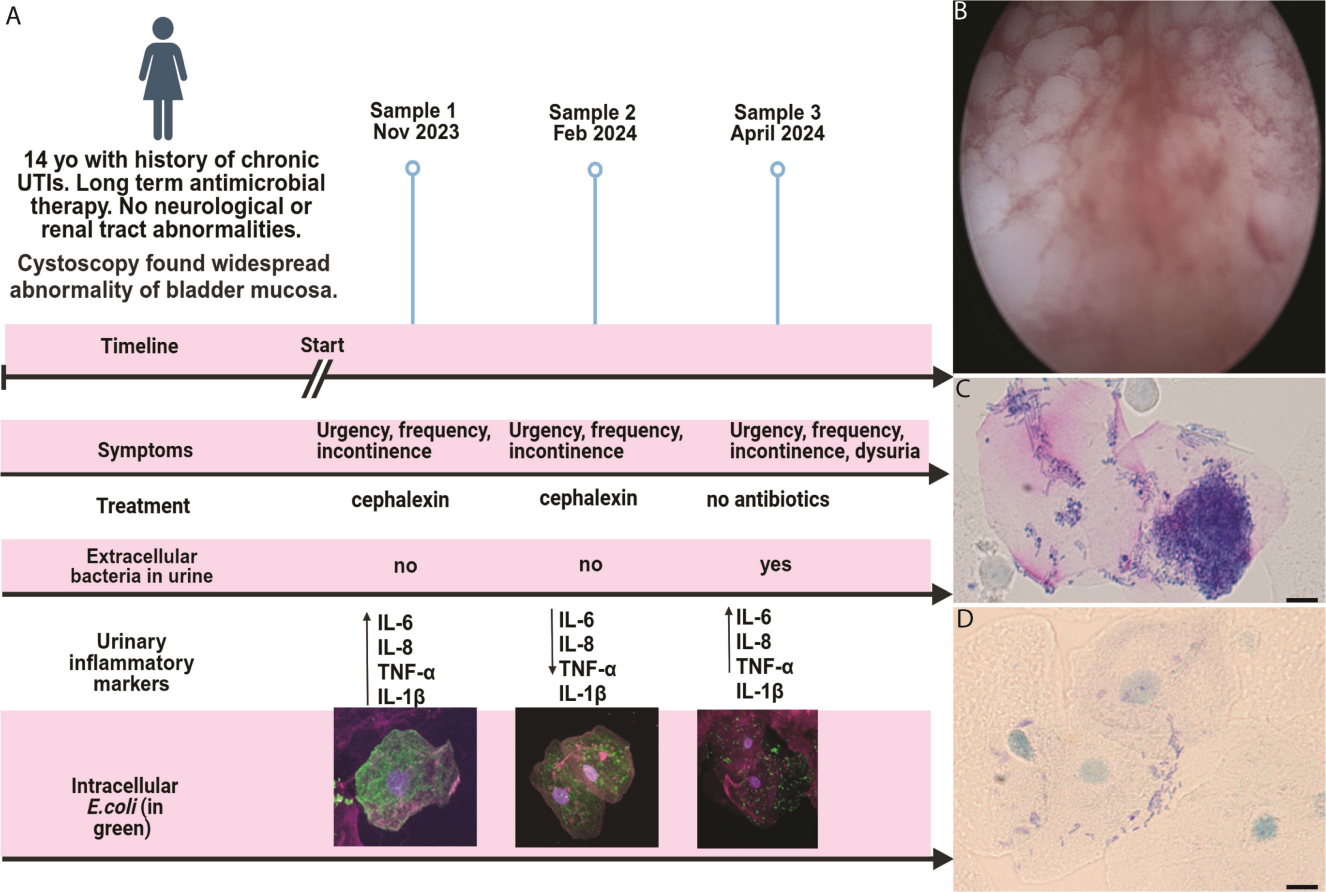
Case summary of a 14-year-old girl with chronic UTI and persistent IBCs. (**A**) Patient symptoms, treatment timeline, and corresponding findings from urine samples. Research samples were tested for intracellular *E. coli* using microscopy, with urinary cytokine quantification. Some of the IBC images are mini versions of images displayed in Fig. 2, in greater detail. (**B**) Cystoscopy, done November 2023, identified extensive keratinizing squamous metaplasia of the bladder mucosa. (**C and D**) Desquamated epithelial cells demonstrated extensive IBCs under spinning disk confocal microscopy (stained in blue with Wright stain), displaying different phenotypes. Created with BioRender.com. IBC, intracellular bacterial community; UTI, urinary tract infection.

At presentation in early November 2023 (Fig. 1A), urine microscopy demonstrated pyuria with mid-stream urine cultures positive for pan-susceptible *E. coli* (at 10^8^ CFU/L) and a white blood cell count of 1 × 10^8^ cells/L. The *E. coli* cleared from her urine following a 7-day course of cephalexin (15 /mg/kg), but symptoms recurred within days of completing antibiotic therapy. Hence, in late November 2023, a urine sample was collected to explore the presence of intracellular *E. coli* as a possible explanation for her recurrent positive urine cultures and persistent UTI symptoms.

A cystoscopy confirmed widespread non-keratinizing squamous metaplasia of the bladder mucosa or urothelium (Fig. 1B). Bladder wall histology showed gram-negative rods and biopsy cultures identified as pan-sensitive *E. coli*. She subsequently received a 6-month course of cephalexin to try and break the cycle of chronic infection, with ongoing clinical review and urine sampling to assess for intracellular bacteria (IBCs). Following completion of cephalexin, she was placed on prophylactic trimethoprim-sulfamethoxazole with D-mannose supplementation to try and reduce the risk of bacterial adhesion.

### Persistent IBCs in exfoliated urothelial cells

**Fig 2:**
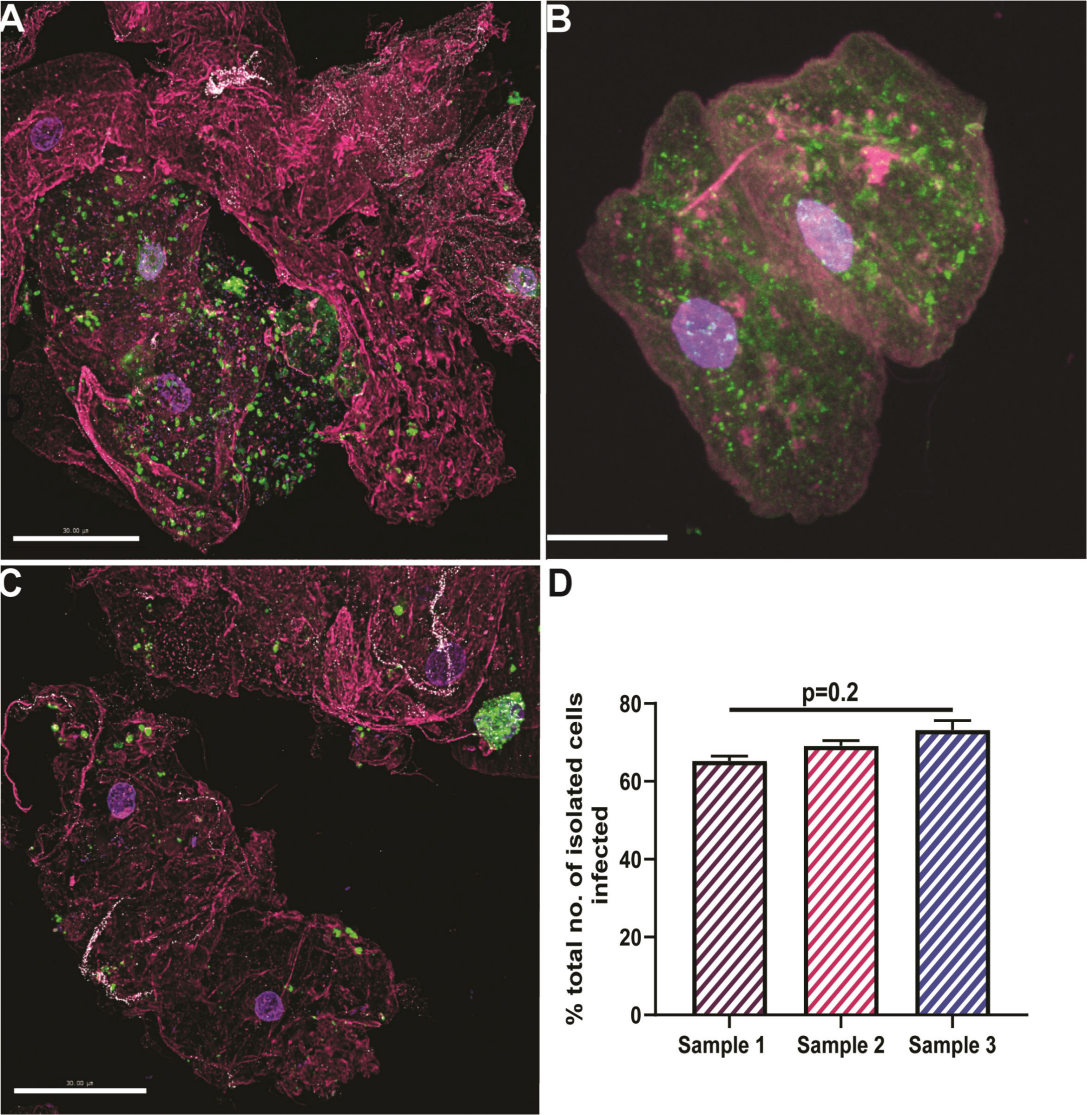
Spinning disk confocal microscopy of urothelial cells with intracellular *E. coli*. Samples were stained with antibodies against *E. coli* (green), uroplakins (white), wheat germ agglutinin (pink), and 4′,6-diamidino-2-phenylindole for the cell nucleus (purple). Representative images of urothelial cells isolated from urine cultures in (**A**) November 2023 (sample 1), (**B**) January 2024 (sample 2, also shown in Fig. 1), and (**C**) April 2024 (sample 3). Scale bar denotes 50 μm. (**D**) Percentage of imaged exfoliated cells with IBCs across the three samples. One-sample Wilcoxon *t*-test was used to assess statistical significance as indicated. IBC, intracellular bacterial communities.

**Fig 3:**
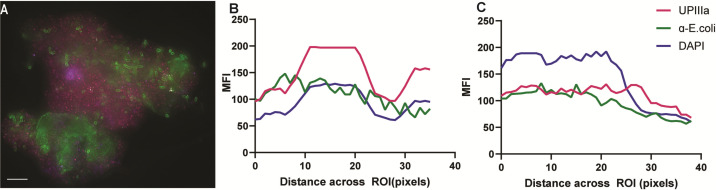
*E. coli* localization in desquamated epithelial cells. IBCs were imaged using SDCM with subsequent quantification. SDCM image depicting UPEC biofilm communities (green) in cells stained with UPIIIa (pink) (**A**). A representative MFI plot shows the location of the UPEC signal relative to the UPIIIa and DAPI signal along the longitudinal ROI along the *y*-axis, as well as a cross-section slice on the average taken from different representative images (**B and C**). No. of ROIs analyzed per sample = 40. Scalebar = 10 micrometres. DAPI, 4′,6-diamidino-2-phenylindole; IBC, intracellular bacterial community; MFI, mean fluorescence intensity; ROI, region of interest; SDCM, spinning disk confocal microscopy; UPIIIa, uroplakin 3a.

Mid-stream urine samples were collected at 3-month intervals for a 6-month period for high-resolution spinning disk confocal microscopy (SDCM) and expanded testing. Cells were stained for anti-UPIIIa overnight (1/100 dilution; Abcam, USA) followed by fluorescein isothiocyanate-conjugated anti-*E*. *coli* (1/500 dilution, Abcam), Alexa-Fluor 647-conjugated wheat germ agglutinin (Thermo Fisher Scientific, Australia), and 4′,6-diamidino-2-phenylindole (Thermo Fisher Scientific). Using SDCM (3i Multimodal microscope (Intelligent Imaging Innovations, Inc., Denver, CO, USA), the presence of extensive IBCs was consistently observed in exfoliated urothelial cells over a 6-month period (Fig. 2A through C), usually in the presence of intracellular bacterial biofilm aggregates. This occurred despite prolonged antibiotic therapy and in the absence of positive urine cultures (for extracellular *E. coli* in two consecutive samples tested 3 months apart) (Fig. 1A). However, *E. coli* was isolated from these samples once infected urothelial cells were lysed using 1%Triton X-100 at 4°C for 20 minutes. 3-D reconstructions of the SDCM images show dense bacterial communities heterogeneously distributed within the infected cells (Fig. 2A through C; Fig. 3A through C).

**Fig 4:**
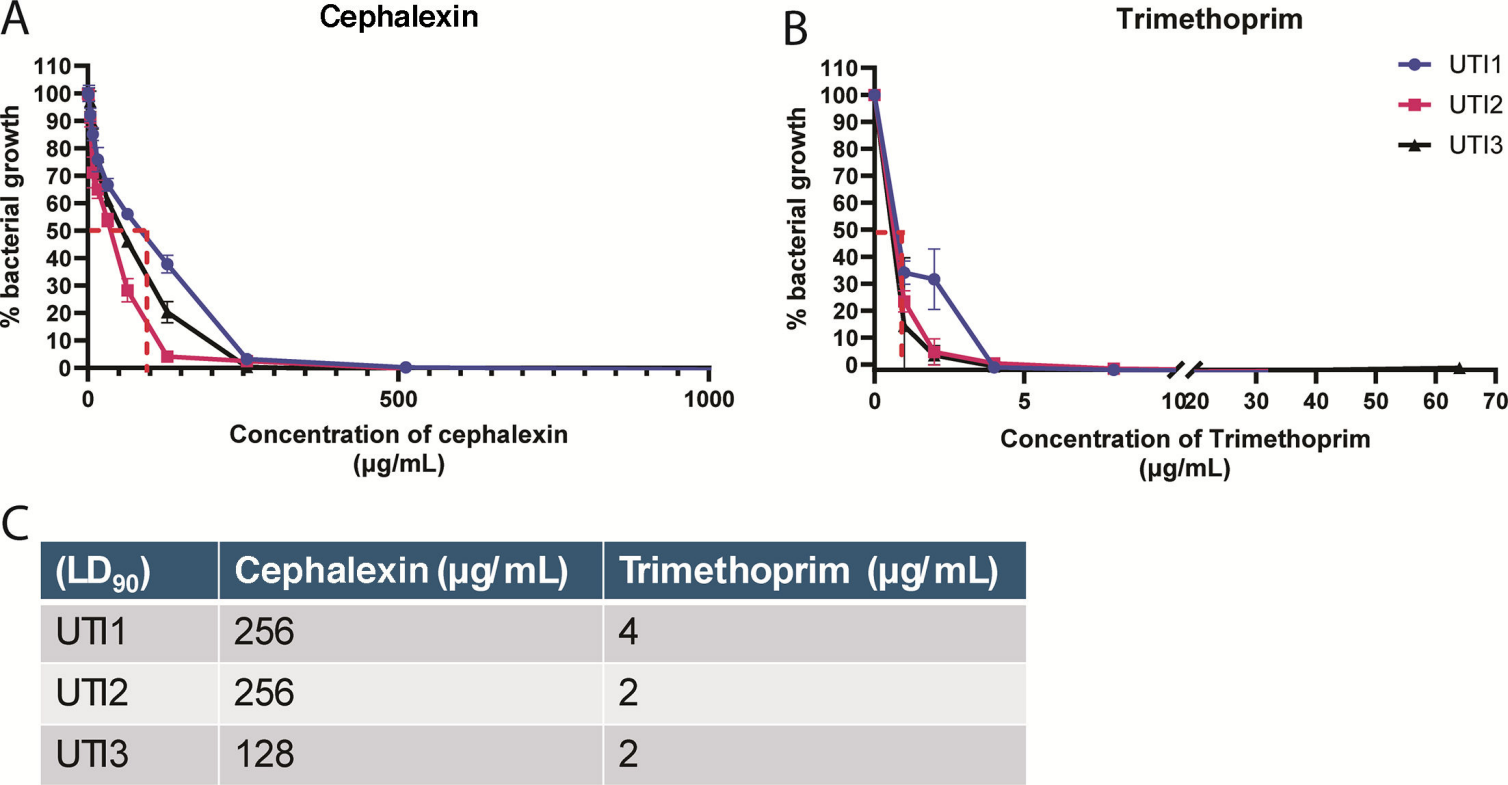
MICs determined for the UPEC strains isolated from each sample. Microdilution assays were performed on UPEC isolates from the three research samples according to EUCAST guidelines to determine the MICs of isolated *E. coli* strains to (**A**) cephalexin and (**B**) trimethoprim. (**C**) MIC_90_ (concentration at which 90% of bacteria are killed) for both antibiotics. UTI1 and UTI2 were isolated from intracellular reservoirs in urothelial cells. UTI3 was isolated from extracellular urine culture. MIC, minimum inhibitory concentration.

Over 60% (50/80) of urothelial cells imaged were positive for *E. coli* (40 regions/sample), 65% (82/120) in sample 1, and 75% (69/92) in sample 3. No significant differences were present in the intracellular bacterial loads quantified in the three samples (*P* = 0.2) (Fig. 2D). Although standard urine isolates were fully drug susceptible, the three isolates grown from intracellular bacteria during the study were found to be resistant to cephalexin (well over the European Committee on Antimicrobial Susceptibility Testing [EUCAST] clinical breakpoints of 16 mg/L) (Fig. 4A and C) but susceptible to trimethoprim (Fig. 4B and C).

### High urinary inflammatory cytokine levels

**Fig 5:**
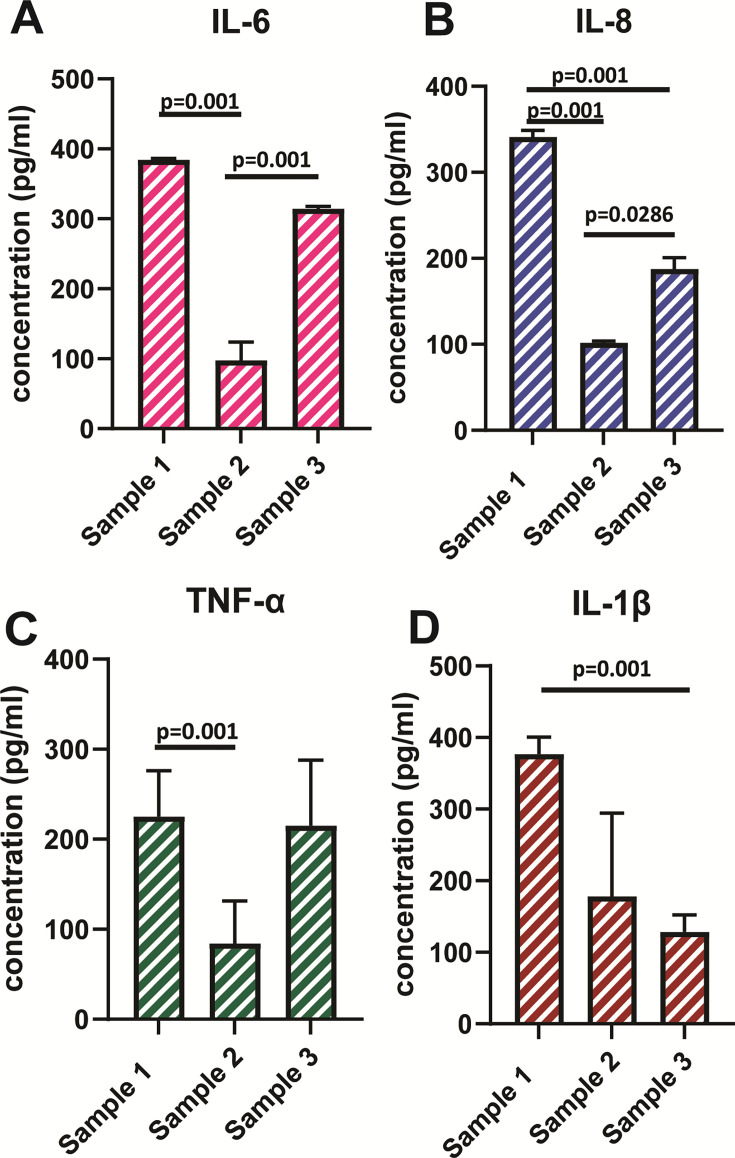
Urinary cytokine profile in a 14-year-old girl with chronic UTI over a 6-month period. Enzyme-linked immunosorbent sandwich assays were done on freshly collected urine samples to quantify key urinary cytokines, with each sample performed in triplicate. Comparisons were drawn between levels of IL-6, IL-8, TNF-α, and IL-1β (**A–D**) in urine samples obtained 3 months apart. Dunnett’s T3 multiple comparison tests were used to assess statistical significance for relevant comparisons, as indicated.

Urinary inflammatory cytokines remained high, independent of IBC presence or antibiotic treatment. Sample 1 yielded almost threefold higher levels of IL-6 (80 pg/mL), IL-8 (325 pg/mL), and tumor necrosis factor alpha (TNF-α) (225 pg/mL) compared to sample 2 obtained 3 months later (*P* < 0.02). However, sample 3 contained interleukin (IL)-6, IL-8, and TNF-α levels at similar levels to sample 1 (Fig. 5A through C). IL-1β levels showed an opposite trend, with sample 2 yielding a higher concentration compared to sample 1 (*P* < 0.02) (Fig. 5D), with sample 3 showing a continuing reduction in IL-1β concentration, almost four times lower than the first sample.

## DISCUSSION

Our report is the first to identify long-term IBCs in a child with chronic UTI, despite prolonged antimicrobial treatment and documented UTI clearance, using routine culture and microscopy. The persistence of IBCs, in the presence of negative urine cultures during routine testing, has not been previously reported. Our study demonstrates that IBCs can persist despite antibiotic treatment and that standard urine testing may falsely suggest urinary clearance if IBCs are not specifically tested for.

The formation of IBCs provides a mechanism by which UPEC and other uropathogens create reservoirs that evade immune attack, which may facilitate disease relapse (13, 14). The squamous metaplasia observed on cystoscopy is likely a response to chronic inflammation of the bladder wall, triggered by unresolved infection (15). It is uncertain whether the presence of squamous metaplasia facilitates IBC formation or vice versa and what the longer-term sequelae of squamous metaplasia may be.

The lack of culturable extracellular bacteria suggests effective antibiotic treatment of the infected urine but also highlights a critical gap in diagnostic urine culture methods that fail to detect IBCs. Both SDCM and standard brightfield microscopy were used to detect the presence of both rod- and coccus-shaped UPEC associated with and within cells, typical of the morphological plasticity the bacteria undergo to promote long-term persistence and avoid immune response activation (16). The lack of culturable extracellular bacteria in planktonic and filamentous forms, which is often found in the urine of patients with IBCs, suggests effective antibiotic treatment of the infected urine despite the documented cephalexin resistance detected in intracellular bacteria.

Beta-lactam antibiotics that are renally excreted, like cephalexin, achieve very high urinary levels (17) that often exceed moderate increases in bacterial MIC and may still provide effective UTI treatment. However, in this instance, the IBC bacteria were unaffected and displayed MICs that greatly exceeded EUCAST thresholds. It has been demonstrated that cell membrane-permeable antibiotics such as ciprofloxacin achieve superior clearance of IBCs in *in vitro* and animal models (18, 19). However, a deep understanding of which antibiotics penetrate human host cells best, to clear IBCs, is lacking. Current antibiotic adjunct therapies include D-mannose, which prevents UPEC binding to the epithelial cells and methamine hippurate, which acidifies the urine. These treatments may have prophylactic value but cannot eradicate established IBCs. Treatments such as protamine sulfate and forskolin have been used for experimental IBC expulsion, while immunomodulation using COX-2 inhibition has shown promise to reduce bladder inflammation in mouse models (14, 20) but its value in humans with an inflamed and irritable bladder has not been documented.

Relapse from IBC reservoirs likely explains the rapid recurrence of symptoms and positive cultures after antibiotic discontinuation. Whole genome sequencing could confirm if relapse involves identical bacteria or a multistrain infection. Another possible source of resistance to antibiotic treatment in the IBC bacteria may be the presence of bacterial L-forms. Both confocal microscopy and brightfield microscopy were used to detect the presence of rod- and coccus-shaped UPEC within cells. This morphological plasticity promotes long-term persistence and avoids immune response activation (16). L-forms are a cell wall-deficient state adopted by bacteria during antibiotic exposure, rendering them resistant to cell wall-acting antibiotics, thus possibly explaining the high MICs observed (21). L-forms are an understudied phenomenon, and more work is required to explore their potential presence and relevance in patients.

Another interesting finding from this study was the highly elevated and fluctuating levels of urinary cytokines, markedly above healthy control reference ranges (22–24). In mouse models, high levels of IL-6, IL-5, IL-8, and granulocyte-macrophage colony-stimulating factor (GM-CSF) are predictive of ensuing infection (25). In mice with TNF-α depletion, the level of bacterial invasion and urothelium damage was significantly attenuated (26), suggesting that urinary TNF-α levels may influence IBC persistence. To date, there has been no unique relationship described between urinary cytokine levels and the presence of IBCs. We suspect that patients with IBCs might have unique cytokine profiles compared to patients with general UTIs, warranting larger wide-scale investigations. The inverse correlation between IL-1β and other cytokines is of special interest, as IL-1β is responsible for the production of inducible nitric oxide synthase (iNOS) and nitric oxide (NO) with upregulation of the caspase pathway, which can result in enhanced exfoliation and urothelium destruction, as observed in our patient. This study reignites the importance of pursuing cytokine profiles as potential non-invasive diagnostic biomarkers of persistent infection.

This is the first documented case of long-term IBCs in a patient with persistent symptoms. Current diagnostic and treatment standards for recurrent and/or chronic UTIs may not detect persistent IBC reservoirs and provide ineffective antibiotic therapy. This highlights the critical unmet need for the development of specific diagnostics that can detect the presence of IBCs. Moreover, patients with persistent IBCs must be treated as distinct from those with uncomplicated recurrent UTIs, given the differences in underlying pathology and treatment. This case study provides a template for future studies on severely symptomatic young females whose UTI symptoms are only temporarily alleviated by standard treatments.

Understanding the pathogenesis of persistent IBCs in children with chronic UTIs is crucial to guide optimal therapy. The patient at the time of writing (April 2025) is still on antibiotic therapy, supplemented with non-antibiotic treatments such as D-mannose. She still shows evidence of IBC colonization with no solution in sight. Persistent IBCs may act as bacterial reservoirs, driving relapse, antimicrobial resistance, and chronic bladder inflammation, leading to irritability and urothelial metaplasia. Our case highlights the urgent need to explore the mechanisms and immune responses behind severe chronic UTIs, which is a particular problem in post-partum women but also in some female adolescents.
